# Radial shortening following a fracture of the proximal radius: Degree of shortening and short-term outcome in 22 proximal radial fractures

**DOI:** 10.3109/17453674.2011.574563

**Published:** 2011-07-08

**Authors:** Andrew D Duckworth, Bruce S Watson, Elizabeth M Will, Brad A Petrisor, Phillip J Walmsley, Charles M Court-Brown, Margaret M McQueen

**Affiliations:** ^1^Edinburgh Orthopaedic Trauma Unit; ^2^SORT-IT, Edinburgh Royal Infirmary, Edinburgh, UK; ^3^Orthopaedic Trauma Service, Hamilton General Hospital, Hamilton, ON, Canada; ^4^Department of Trauma and Orthopaedic Surgery, Queen Margaret Hospital, Dunfermline, UK

## Abstract

**Background and purpose:**

The Essex-Lopresti lesion is thought to be rare, with a varying degree of disruption to forearm stability probable. We describe the range of radial shortening that occurs following a fracture of the proximal radius, as well as the short-term outcome in these patients.

**Patients and methods:**

Over an 18-month period, we prospectively assessed all patients with a radiographically confirmed proximal radial fracture. Patients noted to have ipsilateral wrist pain at initial presentation underwent bilateral radiography to determine whether there was disruption of the distal radio-ulnar joint suggestive of an Essex-Lopresti lesion. Outcome was assessed after a mean of 6 (1.5–12) months using clinical and radiographic results, including the Mayo elbow score (MES) and the short musculoskeletal function assessment (SMFA) questionnaire. One patient with a Mason type-I fracture was lost to follow-up after initial presentation.

**Results:**

60 patients had ipsilateral wrist pain at the initial assessment of 237 proximal radial fractures. Radial shortening of **≥** 2mm (range: 2–4mm) was seen in 22 patients (mean age 48 (19–79) years, 16 females). The most frequent mechanism of injury was a fall from standing height (10/22). 21 fractures were classified as being Mason type-I or type-II, all of which were managed nonoperatively. One Mason type-III fracture underwent acute radial head replacement. Functional outcome was assessed in 21 patients. We found an excellent or good MES in 18 of the 20 patients with a Mason type-I or type-II injury.

**Interpretation:**

The incidence of the Essex-Lopresti lesion type is possibly under-reported as there is a spectrum of injuries, and subtle disruptions often go unidentified. A full assessment of all patients with a proximal radial fracture is required in order to identify these injuries, and the index of suspicion is raised as the complexity of the fracture increases.

The Essex-Lopresti lesion is the eponym given to radio-ulnar instability caused by sequential injury to the distal radio-ulnar joint, the interosseous membrane, and fracture of the proximal radius (Essex-Lopresti 1951). The original paper by Essex-Lopresti suggested that this is a rare injury, with subsequent literature indicating that the lesion is present in approximately 1% of all radial head fractures (Essex-Lopresti 1951, Trousdale et al. 1992). A varying degree of force, in order to sustain such an injury, has been reported (Hotchkiss et al. 1989, Wallace et al. 1997).

The diagnosis and treatment are challenging, with further imaging used when an unstable lesion is suspected (Starch and Dabezies 2001, Wallace 2002). When there is instability, open reduction and internal fixation or radial head replacement with added longitudinal stabilization of the forearm bones is recommended (Lewin 1973, Edwards and Jupiter 1988, Bruckner et al. 1996).

A recent study has shown that the incidence of associated injuries following radial head fractures is high on MRI (Kaas et al. 2010). The purpose of our study was to describe the spectrum of radial shortening suggestive of an Essex-Lopresti lesion that occurs following a radial head or neck fracture, along with determining the short-term outcome in these patients.

## Patients and methods

Our trauma unit treats a defined population of over 500,000, and is the primary acute musculoskeletal trauma service for the local adult population (≥ 13 years of age). Over an 18-month period, we performed a prospective analysis of all patients who presented to our unit with a closed proximal radial fracture (Duckworth et al. 2011). This study focused on a subgroup of these patients who presented with a closed proximal radial fracture and ipsilateral wrist pain. No methods of assessment or intervention were changed at the time of the study, so this was considered to be an audit and it did not require ethical approval according to local guidelines.

**Figure 1.**
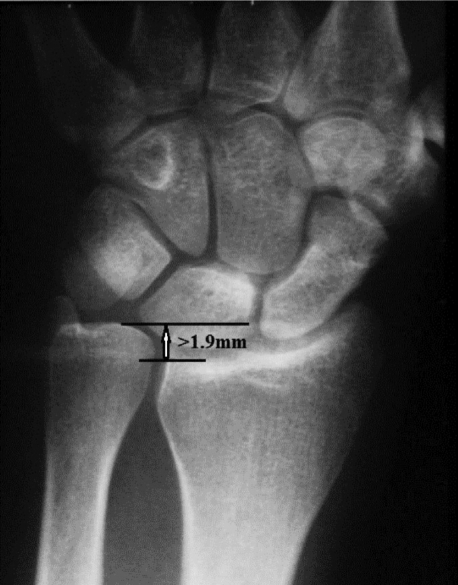
An Essex-Lopresti lesion was diagnosed in this patient's radiographs, which demonstrated greater than 2 mm of shortening of the ipsilateral radius when compared to the contralateral side.

Radiographic assessment used standard anteroposterior (AP) and lateral radiographs of the elbow. Initial elbow radiographs were used to classify the proximal radial fracture using the modified Mason (Broberg and Morrey 1987) classification system. If the patient had wrist pain or tenderness, neutral PA and lateral radiographs of the affected wrist were performed to screen for the presence of any distal radial-ulnar joint abnormality. Radiographs of the unaffected contralateral wrist were performed for comparison. If there was 2 mm or more of ipsilateral radial shortening compared to the contralateral side, this was considered to be suggestive of an Essex-Lopresti type lesion (Figure 1). 2 trauma-trained fellows independently assessed and classified each injury, and any discrepancies were resolved by discussion with the senior authors. All diagnoses were made within 2 weeks of injury.

The final treatment decision for individual patients was determined independently by their supervising consultants, all of whom were experienced orthopedic trauma surgeons. Nonoperative management involved a standardized protocol of a collar and cuff for 1 week, followed by supervised physiotherapy. Physiotherapy included standard mobilization and strengthening exercises of the affected elbow and wrist as symptomatology allowed. Operative management could use either open reduction and internal fixation or radial head replacement, depending on the degree of comminution to the radial head.

### Follow-up

Patients were scheduled for review at 2 and 6 weeks, at 3 and 6 months, and 1 year after injury. Demographic data were recorded at the initial visit and results of physical examination, treatment, and complications were recorded at each subsequent visit. Both clinical and radiographic follow-up were carried out by the senior authors in all cases. At each visit, a full functional assessment was completed by a single dedicated research physiotherapist who was not involved in patient management. The range of motion was measured using a standard full-circle goniometer. Flexion, extension, supination, and pronation in the affected elbow were measured in triplicate and the mean was recorded to minimize intra-observer bias. The Mayo elbow score (MES) and the short musculoskeletal function assessment (SMFA) questionnaire were completed by all patients at each visit (Morrey et al. 1993, Swiontkowski et al. 1999).

### Statistics

SPSS software version 17.0 was used for statistical analysis. The Mann-Whitney U-test was used to analyze non-parametric continuous data. The Kruskal-Wallis one-way analysis of variance test was used to analyze data for several groups. Significance was determined as a p-value of < 0.05 in all analyses, with 95% confidence intervals (CIs) set.

The kappa value was used to determine the inter-observer reliability of diagnosing an Essex-Lopresti type injury as 2 mm or more of ipsilateral radial shortening compared to the contralateral side, with an associated grading assigned as follows: slight agreement (0.00–0.20), fair agreement (0.21–0.40), moderate agreement (0.41–0.60), substantial agreement (0.61–0.80), and very good agreement (≥ 0.81) (Landis and Koch 1977). The 95% CI for the kappa value was calculated using the standard formula, which was kappa value ± 1.96 × the standard error (0.026).

## Results

60 patients were noted to have ipsilateral wrist pain at the initial assessment of 237 proximal radial fractures. Radial shortening of ≥ 2 mm (mean 2.5 (2–4) mm) was seen in 22 patients (9%; CI: 6–14) with a mean age of 48 (19–79) years, 16 of whom were females. There were 2 disagreements regarding the diagnosis of radial shortening between the 2 trauma-trained fellows, giving a kappa value of 0.96 (CI: 0.9–1), with the strength of agreement considered to be very good.

The most frequent mechanism of injury was a fall from standing height (45%) followed by sports-related injuries (27%). There was no association between the mechanism of injury and the degree of radial shortening (p = 0.2). The left elbow was affected in 14 patients and the dominant side in 9 patients. There were 10 radial head fractures and 12 radial neck fractures. Using the Mason classification, there were 13 type-I fractures, 8 type-II fractures, and 1 type-III fracture. The degree of radial shortening varied with increasing fracture severity (p = 0.04) (Table). There were no fractures associated with a dislocation of the elbow.

**Table:** The degree of radial shortening measured on bilateral wrist radiographs, categorized by fracture complexity according to the Mason classification

Mason classification	Age, years median (range)	Radial shortening, mm median (range)
I	39 (19–78)	2.0 (2–3)
II	54 (38–79)	2.5 (2–3.5)
III	31 (NA)	4 (NA)

Nonoperative management was employed in 21 patients. Operative management was employed in 1 patient due to substantial comminution and displacement of the radial head—the one Mason type-III fracture with suspected forearm instability that was confirmed intraoperatively. This patient underwent acute radial head replacement with no complications.

Of the 22 patients who were diagnosed with ≥ 2 mm of radial shortening suggestive of an Essex-Lopresti type lesion, 21 attended a follow-up examination (10 radial head and 11 radial neck; 12 Mason type-I, 8 Mason type-II, and 1 Mason type-III). The mean follow-up was 6 (1.5–12) months.

**Figure 2.**
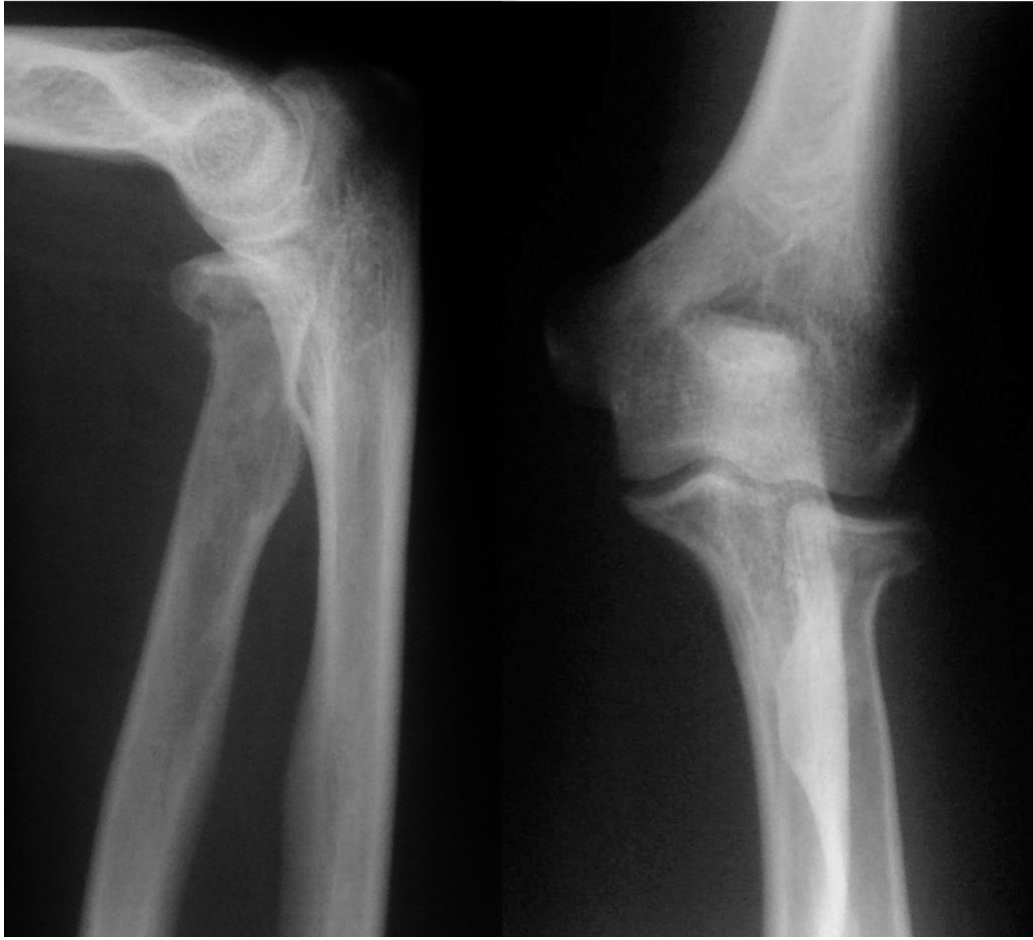
Initial presentation AP and lateral radiographs of a 27-year-old male who sustained a Mason type-I fracture of the left elbow while playing sport. An Essex-Lopresti lesion was diagnosed at 2 weeks when bilateral wrist radiographs were taken. Management was conservative, using collar and cuff immobilization for 1 week followed by supervised physiotherapy. At 6 months, the patient had a flexion arc of 152 degrees and a forearm rotation arc of 180 degrees, with an excellent MES (100).

The mean MES at final evaluation was excellent: 90 (70–100), with a mean flexion arc of 136 (90–154) degrees and a mean forearm rotation arc of 175 (90–180) degrees. The mean SMFA at final evaluation was 4.8 (0–26). 18 patients achieved excellent or good functional results as measured on the MES (Figure 2). Of the 3 patients who did not achieve excellent or good functional results, all had MES values of 70, with 2 patients being treated nonoperatively. 1 patient who was lost to follow-up at 3 months had mild elbow stiffness, but had a flexion arc of 144 degrees and a rotation arc of 180 degrees. 1 patient developed shoulder capsulitis secondarily, delaying recovery. The patient who underwent operative intervention with radial head replacement had a final MES of 70, with notable elbow stiffness.

## Discussion

In this paper, we describe a range of radial shortening suggestive of an Essex-Lopresti lesion following a fracture of the radial head or neck. Due to the small numbers in our study, we were unable to determine the degree of radial shortening found on radiographs that would be indicative of an unstable Essex-Lopresti lesion. However, all patients treated as having a stable lesion had less than 4 mm of radial shortening. Our findings indicate that stable lesions need no treatment. For the unstable Essex-Lopresti injuries, the treatment algorithm described by Edwards and Jupiter (1988) is commonly used.

Recent studies have examined the less complex fractures of the proximal radius and the possible association with Essex-Lopresti lesions (Hausmann et al. 2009, Helmerhorst and Ring 2009). A study examining low-energy Mason type-I radial head fractures with MRI of the forearm revealed partial ruptures of the distal interosseous membrane without disruption of the interosseous ligament (Hausmann et al. 2009). However, they reported that none of their patients had abnormalities on wrist examination or imaging diagnostic for an Essex-Lopresti lesion, which was present in all of our patients. Furthermore, Essex-Lopresti injuries have been described with minimally displaced partial fractures (Mason type-II) of the proximal radius (Helmerhorst and Ring 2009). We found that as the complexity of the fracture increases, the degree of radial shortening also increases; thus, the index of suspicion for instability should increase.

As with isolated proximal radial fractures, the most common mechanism of injury we found was a fall onto an outstretched arm, and no association was found between the mechanism of injury and the degree of radial shortening. The original paper by Essex-Lopresti suggested that the injury was sustained following a “violent longitudinal compression force in the long axis of the radius”, and the mechanism of injury and the force required to cause it has been the focus of many studies since (Essex-Lopresti 1951, McDougall and White 1957). Essex-Lopresti lesions are often suspected following high-energy falls onto an outstretched hand, e.g. a fall from a height (Dodds et al. 2008, Green and Zelouf 2009). However, in the literature, a varying degree of force has been said to be required to disrupt the triangular fibrocartilage complex, the interosseous ligament, and to fracture the radial head (Hotchkiss et al. 1989, Wallace et al. 1997). It has been suggested that the wide range of forces quoted may be related to the position of the forearm at the time of injury (Manson et al. 2000, Markolf et al. 2000, Shepard et al. 2001). We feel that, irrespective of the mechanism of injury, it is essential to perform a full assessment of the forearm to exclude the presence of an Essex-Lopresti lesion.

We routinely use radiographs as our initial line of investigation (Edwards and Jupiter 1988, Jungbluth et al. 2006, Yeh et al. 2001). Radiographs of the wrist should include a “true lateral” radiograph, as well as satisfactory true posterior-anterior views in neutral rotation, to ascertain whether there is subluxation or dislocation of the distal radioulnar joint (Edwards and Jupiter 1988, Yeh et al. 2001). Bilateral comparison enables assessment of the individual's normal ulnar variance on the uninjured side. Ultrasound, CT, and MRI have been shown to provide accurate information regarding the integrity of the interosseous membrane and confirmation of congruency of the distal radioulnar joint (Mino et al. 1983, Starch and Dabezies 2001, Wallace 2002).

The main strength of our study is that it represents a large, unselected series of patients with a proximal radial fracture, with prospective data collection. Our short-term follow-up, with some patients not completing a full year follow-up, along with the small numbers, is a drawback. However, as we are the only primary acute musculoskeletal trauma service for the local adult population, it can be argued that most of these patients did not re-attend as they were asymptomatic. We do not routinely perform an MRI unless instability is suspected. The lack of MRI to confirm the diagnosis might be criticized, however. In some institutions, the availability of such imaging facilities may be limited and might cause delay in the diagnosis and treatment of the patient. Further work in this area using MRI would be beneficial.
